# LncRNA TTN-AS1 promotes the progression of oral squamous cell carcinoma via miR-411-3p/NFAT5 axis

**DOI:** 10.1186/s12935-020-01378-6

**Published:** 2020-08-28

**Authors:** Su-Wei Fu, Yan Zhang, Shen Li, Zhi-Yan Shi, Juan Zhao, Qing-Li He

**Affiliations:** Department of Stomatology, Henan Provincial People’s Hospital, People’s Hospital of Zhengzhou University, No.7 Weiwu Road, Zhengzhou, 450003 Henan China

**Keywords:** TTN-AS1, miR-411-3p, NFAT5, Oral squamous cell carcinoma

## Abstract

**Background:**

Oral squamous cell carcinoma (OSCC) is a common kind of squamous cell carcinoma of the head and neck, which is a threat to public health. Long noncoding RNAs (lncRNAs) are associated with the development of various diseases, including cancers. LncRNA titin antisense RNA 1 (TTN-AS1) is known as a crucial regulatory factor in several cancers. Nevertheless, the specific functions of TTN-AS1 in OSCC remains obscure.

**Methods:**

The expression of TTN-AS1 in OSCC samples or cells was analyzed through qRT-PCR. Colony formation assay, EdU assay, flow cytometry assay, TUNEL assay and wound healing assay were conducted to estimate the functions of TTN-AS1 in OSCC cells. RIP and luciferase reporter assays were utilized to detect the interaction between TTN-AS1 and miR-411-3p as well as between miR-411-3p and NFAT5.

**Results:**

TTN-AS1 expression was stronger in OSCC cells. Knockdown of TTN-AS1 effectively restrained cell proliferation and migration but had inductive role in apoptosis. Moreover, TTN-AS1 could function as the miR-411-3p sponge in OSCC and miR-411-3p exerted the inhibitory functions on OSCC cell growth. In addition, NFAT5 was proven as the target of miR-411-3p. Rescue assay indicated that overexpressing NFAT5 could reverse the inhibitory function of TTN-AS1 depletion on cell growth.

**Conclusion:**

lncRNA TTN-AS1 contributed to the progression of OSCC via miR-411-3p/NFAT5 axis.

## Background

Oral squamous cell carcinoma (OSCC) is one of the commonest squamous cell carcinomas occurs in the head and neck. It ranks sixth in occurrence and had a high mortality rate [1, 2]. According to many years of investigation and research, the pathogenesis of OSCC is related to the internal factors such as drinking and smoking, but its specific pathogenesis is still unclear [1, 3]. Although the surgery for OSCC is effective, the situation for the overall survival of OSCC patients is still unfavorable [4, 5]. Thus, in-depth study of the potential molecular mechanisms of OSCC is of great significance for developing new therapeutic strategies.

Long noncoding RNAs (lncRNAs) are classified as the subgroup member of noncoding RNAs family with over 200 nucleotides in length, which are not able to encode proteins [6, 7]. Recently, lncRNAs are confirmed to involve in different cell progression, such as cell proliferation and cell apoptosis. Moreover, the crucial functions of lncRNAs in the occurrence and development of assorted cancers have also been reported through a flow of researches [8, 9]. Different kind of lncRNAs exerted different functions in cancers. For example, PVT1 accelerated esophageal carcinoma cell migration and invasion via sponging miR-145 and regulating FSCN1 [10]. SARCC alters he androgen receptor/miRNA-143-3p signals, thereby suppresses the progression of renal cell carcinoma [11]. And GAPLINC facilitated gastric cancer cell growth through serving as a sponge of miR-378 to regulate MAPK1 [12]. Titin antisense RNA 1 (TTN-AS1) is a novel lncRNA that takes part in the regulation of cancer development in accordance with existing researches. For illustration, TTN-AS1 with high expression in lung adenocarcinoma cells can expedite cellular functions of lung adenocarcinoma through serving as a sponge of miR-142-5p to regulate CDK5 [13]. Nevertheless, its specific function of TTN-AS1 in OSCC remains unclear.

Here, we selected TTN-AS1 as the object of our research and investigated the regulatory mechanisms and functions in OSCC.

## Methods

### Tissues samples

Paired tissues (adjacent normal and tumor) were collected from 50 patients with OSCC who were diagnosed at Henan Provincial People’s Hospital. Patients participated in this study didn’t receive any kind of therapy before surgery. All patients enrolled in this study had signed informed consent. This study received the approval of the Ethics Committee of Henan Provincial People’s Hospital. Samples were stored at − 80 °C until use.

### Cell lines

Human normal squamous epithelial cell line (NOK) obtained from Shanghai Honsun Biological Technology Co.,Ltd (Shanghai, China), human tongue squamous carcinoma cell lines, including SCC-4, SCC-9, CAL-27 procured from ATCC (Manassas, VA, USA) and BICR-16 cell obtained from European Collection of Authenticated Cell Cultures (ECACC, UK) were used in current study. NOK cell was cultured in DMEM (Gibco, Rockville, MD, USA) with 1% antibiotics and 10% FBS (Gibco). CAL-27 cell was cultured in DMEM containing 10% FBS. SCC4 cell was cultured in DMEM: F12 Medium containing 400 ng/ml hydrocortisone and 10% FBS. SCC-9 cell was cultured in DMEM: F12 Medium containing 2.5 mM l-glutamine, 1.2 g/L sodium bicarbonate, 15 mM HEPES, 0.5 mM sodium pyruvate supplemented with 400 ng/ml hydrocortisone and 10% FBS. BICR-16 cell was cultured in DMEM with 500ug/ml G418 and 10% FBS. Cell culture was conducted under a condition with 5% CO_2_ and 37 °C.

### Total RNA extraction and qRT-PCR

TRIzol Reagent (Invitrogen, Carlsbad CA) was responsible for total RNA extraction from samples or cells. Afterwards, RNA samples were converted into cDNA by employing Reverse Transcriptase Kit (Takara, Shiga, Japan). PowerUp™ SYBR^®^ Green Master Mix (Life Technologies, Grand Island, NY, USA) was utilized for PCR analysis [14]. After amplification, 2^−ΔΔCt^ method was applied to quantify PCR products. U6 snRNA or GAPDH was used as the internal control for lncRNA, mRNA or miRNA. All primers used in this experiments were provided in Additional file 1: Table S1. Each samples were assayed for more than triplicate.

### Transfections

The shRNAs designed for TTN-AS1 or NFAT5, and nonspecific shRNAs, as well as pcDNA3.1-NFAT5 and empty vector, theses transfection plasmids were procured from GenePharma (Shanghai, China). In addition, the miR-411-3p mimics/inhibitor and NC mimics/inhibitor were procured from Genechem (Shanghai, China). SCC-4 and SCC-9 cells were collected for 48 h of plasmid transfections, by use of Lipofectamine 3000 (Invitrogen). Sequence for all plasmids used in current study were listed in Additional file 1: Table S1. Each samples were assayed for more than triplicate.

### CCK-8 assay

As previously described [15], CCK-8 Kit (Beyotime, Shanghai, China) was applied to detect cell viability under manufacturer’s protocols. Cells (5000 cells/well) were planted in 96-well plates. After 24, 48, 72 and 96 h, the CCK-8 reagents were added into each well. Cell viability was detected using a microplate reader to measure the absorbance at the wave length of 450 nm. Each samples were assayed for more than triplicate.

### Colony formation assay

After indicated transfections, SCC-4 and SCC-9 cells were planted into 6-well plates with 500 cells in each well. Following 14-day of cell culture, the resulting colonies were fixed using 4% PFA for 30 min, stained using 0.5% crystal violet solution for 5 min, and finally counted manually [16]. Each samples were assayed for more than triplicate.

### EdU assay

EdU assay was undertaken in cells of SCC-4 and SCC-9 for cell proliferation detection, by use of BeyoClick™ EdU Cell Proliferation Kit (Beyotime, Shanghai, China) with Alexa Fluor 594 [17]. The DAPI staining solution was acquired from Beyotime for detecting cell nucleus. After washing in PBS, cells were studied using inverted microscope (Olympus, Tokyo, Japan). Each samples were assayed for more than triplicate.

### Flow cytometry

Cell apoptosis of transfected SCC-4 and SCC-9 cells was assayed employing the flow cytometer (BD Biosciences, Franklin Lakes, NJ), in the presence of Annexin V/PI double staining kit (Invitrogen). Cell samples were collected from 6-well plates via centrifugation, then stained in Binding Buffer and assayed with flow cytometry [18]. Each samples were assayed for more than triplicate.

### TUNEL assay

The transfected cell samples of SCC-4 and SCC-9 were washed employing PBS and fixed using 4% PFS for TUNEL assay [19], in the presence of TUNEL assay reagent (Merck KGaA, Darmstadt, Germany). Following addition of DAPI staining solution, cell samples were analyzed using optical microscopy (Olympus). Each samples were assayed for more than triplicate.

### Wound healing

The transfected cell samples of SCC-4 and SCC-9 were seeded in 6-well plates and cultivated until 100% confluence [20]. Then, the artificial wounds were created with 200 μL of pipette tip. At 0 and 24 h after incubation in serum-free medium, the distance of wound healing were imaged under microscope (Olympus). Each samples were assayed for more than triplicate.

### Subcellular fractionation

The TTN-AS1 content in cytoplasmic and nuclear fractions of SCC-4 and SCC-9 cells was studied by use of PARIS™ Kit (Invitrogen), as requested by provider. Cell samples were lysed with cell fractionation buffer and cell disruption buffer, then centrifuged for separating cell cytoplasm and cell nucleus [21]. For quantification, GAPDH and U6 served as the cytoplasmic indicator and nuclear indicator, respectively. Each samples were assayed for more than triplicate.

### FISH

The subcellular location of TTN-AS1 in SCC-4 and SCC-9 cells was also studied with FISH assay using the deigned specifically TTN-AS1-probe (Ribobio, Guangzhou, China). After fixation, the digested cells were air-dried and cultured with probes in the hybridization buffer, then treated in DAPI staining buffer [22]. Olympus fluorescence microscope was used for imaging. Each samples were assayed for more than triplicate.

### RNA immunoprecipitation (RIP)

Applying the Magna RIP™ RNA-Binding Protein Immunoprecipitation Kit [23], RIP assay was conducted for RNA interaction in SCC-4 and SCC-9 cells, as guided by provider (Millipore, Bedford, MA). RIP lysis buffer (Thermo Fisher Scientific, Waltham, MA, USA) was applied to obtain the lysates. Lysis was incubated with the magnetic beads (Invitrogen, Carlsbad, CA, USA) conjugated with anti-Ago2 antibody or anti-IgG antibody at 4 °C overnight. Complex was washed and purified according to the protocol of RIP kit used in this experiment. The enrichment of RNAs were examined via RT-qPCR. Each samples were assayed for more than triplicate.

### Luciferase reporter assay

TTN-AS1 fragment covering wild-type or mutant miR-411-3p binding sites were employed to construct TTN-AS1-WT or TTN-AS1-Mut vectors, by use of the pmirGLO dual-luciferase vectors (Promega, Madison, WI). SCC-4 and SCC-9 cells were co-transfected with miR-411-3p mimics or NC mimics, and TTN-AS1-WT or TTN-AS1-Mut vectors for 48 h, followed by analysis of dual-luciferase reporter assay system (Promega) [24]. Renilla luciferase activity was used as the internal control. Each samples were assayed for more than triplicate.

### Western blot

Cells were lysed via RIPA buffer. BCA Protein Assay kit (Pierce Biotechnology, Rockford, IL) was used to assess the concentration of protein. Separation of equal amount of proteins was conducted via 12% SDS-PAGE (Bio-Rad Laboratories, Hercules, CA) followed by the transformation to PVDF membranes (Millipore, Bedford, MA). The membranes were blocked with 5% skim milk and incubated with primary and secondary antibodies. All antibodies were obtained from Abcam (Cambridge, MA, USA). Protein bands were detected using a ECL detection kit (Pierce Biotechnology, Rockford, IL). Each samples were assayed for more than triplicate.

### Animal study

Six 4-week-old BALB/c nude mice (Shanghai Laboratory Animal Center) was subjected to animal study in line with the ethical standards and guidelines of Henan Provincial People’s Hospital. SCC-6 cells (1 × 10^6^) stably transfected with sh-NC or sh-TTN-AS1#1 were injected into the right dorsal flanks of six mice. Tumor sizes and volume were monitored by a caliper every 4 days. Four weeks later, the mice were killed followed with the resection of tumors for measuring tumor weight.

### Statistical analyses

Data of three or more independent assays were exhibited as the mean ± SD. In addition, Student’s t-test or one-way/two-way ANOVA followed by Tukey post hoc test was employed for comparing the group difference, by use of GraphPad Prism 7^®^ (GraphPad Software, Inc., La Jolla, CA, USA). Experimental data were collected when p < 0.05.

## Results

### Knockdown of TTN-AS1 restrains the proliferation and migration of OSCC cells

**Fig. 1:**
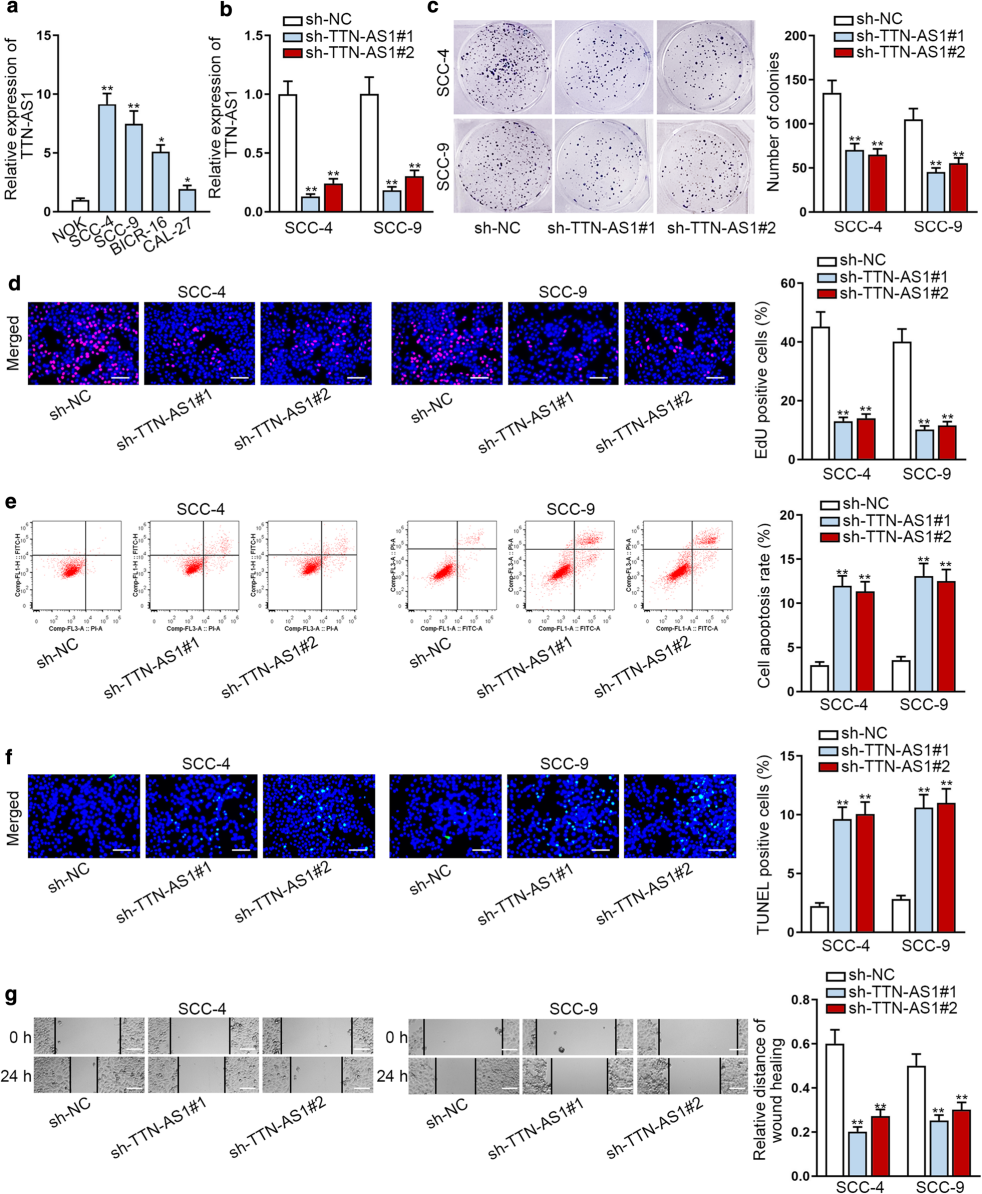
Knockdown of TTN-AS1 restrains the proliferation and migration of OSCC cells. **a** The expression of TTN-AS1 was tested through qRT-PCR in OSCC cells. **b** The interference efficiency of TTN-AS1 was detected by qRT-PCR in SCC-4 and SCC-9 cells. **c**, **d** Cell proliferation ability was measured by colony formation and EdU experiments when TTN-AS1 was inhibited. **e**, **f** Cell apoptosis was evaluated through flow cytometry and TUNEL experiments after silencing TTN-AS1. **g** Wound healing assays were utilized to estimate cell migration when TTN-AS1 was subjected to knockdown. *P < 0.05; **P < 0.01

At first, the relative higher level of TTN-AS1 was observed in OSCC samples rather than adjacent normal ones (Additional file 2: Fig. S1A). Next, we detected the expression of TTN-AS1 in OSCC cells through qRT-PCR analysis. We discovered that TTN-AS1 expression was extremely high in OSCC cells in comparison of normal human squamous epithelial cell (NOK cell) (Fig. 1a). At the same time, we also found that TTN-AS1 expression in SCC-4 and SCC-9 cells was highest. Thus we knocked down TTN-AS1 expression in SCC-4 and SCC-9 cells and identified that the TTN-AS1 expression was exactly declined (Fig. 1b). Following, functional experiments were implemented to test the influence of inhibiting TTN-AS1 on cells proliferation, apoptosis and migration. CCK-8 assay unveiled that TTN-AS1 depletion had significantly suppressive effect on cell viability (Additional file 2: Fig. S1B). The number of colonies and EdU positive cells were reduced after silencing TTN-AS1, indicating that cell proliferation could be restrained by TTN-AS1 depletion (Fig. 1c, d). Then it was found by flow cytometry and TUNEL experiments that apoptosis was accelerated when decreased the level of TTN-AS1 (Fig. 1e, f). Finally, wound healing assay revealed that the migrated capability of SCC-4 and SCC-9 cells was hampered by silencing TTN-AS1 (Fig. 1g). In a word, knockdown of TTN-AS1 restrained cell proliferation and migration of OSCC.

### TTN-AS1 acts as miR-411-3p sponge in OSCC

**Fig. 2:**
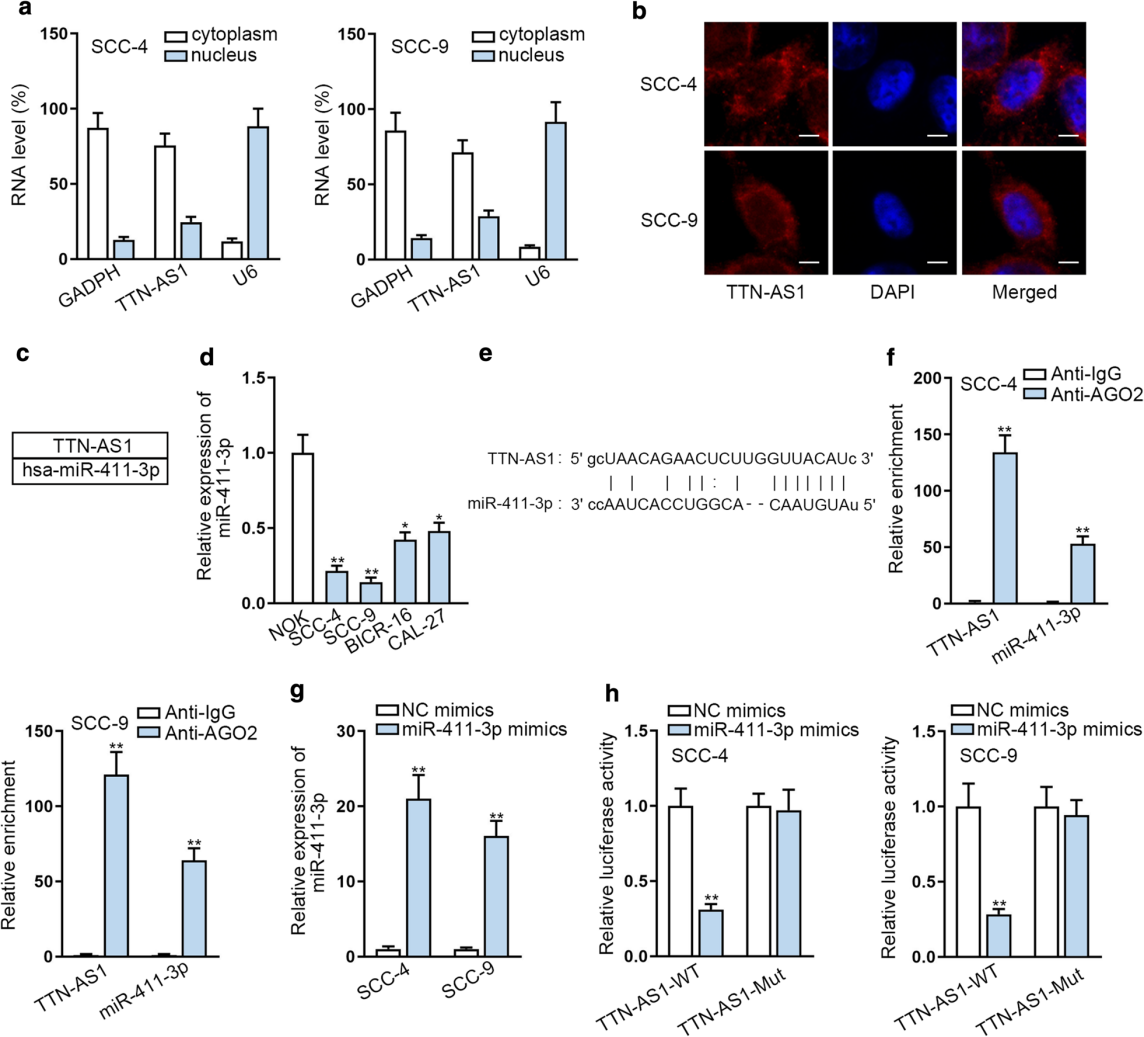
TTN-AS1 acts as the miR-411-3p sponge in OSCC. **a**, **b** The cellular location of TTN-AS1 was identified in SCC-4 and SCC-9 through Subcellular fractionation and FISH. **c** StarBsae website was utilized to predict the possible miRNAs that could bind with TTN-AS1. **d** MiR-411-3p expression was detected through qRT-PCR in OSCC cells. **e** The binding site of TTN-AS1 in miR-411-3p. **f** RIP assay was utilized to evaluate the relationship between miR-411-3p and TTN-AS1. **g** The efficiency of miR-411-3p overexpression was tested through qRT-PCR. **h** Luciferase reporter assays were conducted to verify the correlation of miR-411-3p and TTN-AS1. *P < 0.05; **P < 0.01

Then, we tested the distribution of TTN-AS1 in SCC-4 and SCC-9 cells. The results indicated that TTN-AS1 tended to be located in the cytoplasm of SCC-4 and SCC-9 cells (Fig. 2a, b), indicating the potential post-transcriptional regulatory role of TTN-AS1 in OSCC. A flow of evidence suggested that lncRNA could serve as a ceRNA to regulate mRNAs through sponging miRNAs at post-transcriptional level [25, 26]. Then we utilized starBase website to predict the possible miRNA which could have the binding site of TTN-AS1, and one potential miRNA (miR-411-3p) was found out (Fig. 2c). Then qRT-PCR analysis was implemented to test the expression of miR-411-3p in OSCC samples and cells. And the results indicated that miR-411-3p expression was lower in OSCC tissues and cells (Additional file 2: Fig. S1C and Fig. 2d). The lowest level of miR-411-3p was detected in SCC-4 and SCC-9 cells. After that, we discovered the binding site of miR-411-3p and TTN-AS1 from starBase website (Fig. 2e) and conducted Ago2-RIP assay to evaluate the binding possibility of them. We discovered that miR-411-3p and TTN-AS1 were markedly enriched in anti-Ago2 group (Fig. 2f and Additional file 2: Fig. S1D), which indicated that they were coexisted in RISC. Following, we overexpressed miR-411-3p and conducted the luciferase reporter assay. We discovered that miR-411-3p overexpression caused a notable reduction on the luciferase activity of TTN-AS1-WT while the luciferase activity of TTN-AS1-Mut displayed no visible change (Fig. 2g, h), indicating that TTN-AS1 could bind to miR-411-3p. Overall, TTN-AS1 sponges miR-411-3p in OSCC.

### Upregulation of miR-411-3p represses OSCC cell growth and migration

**Fig. 3:**
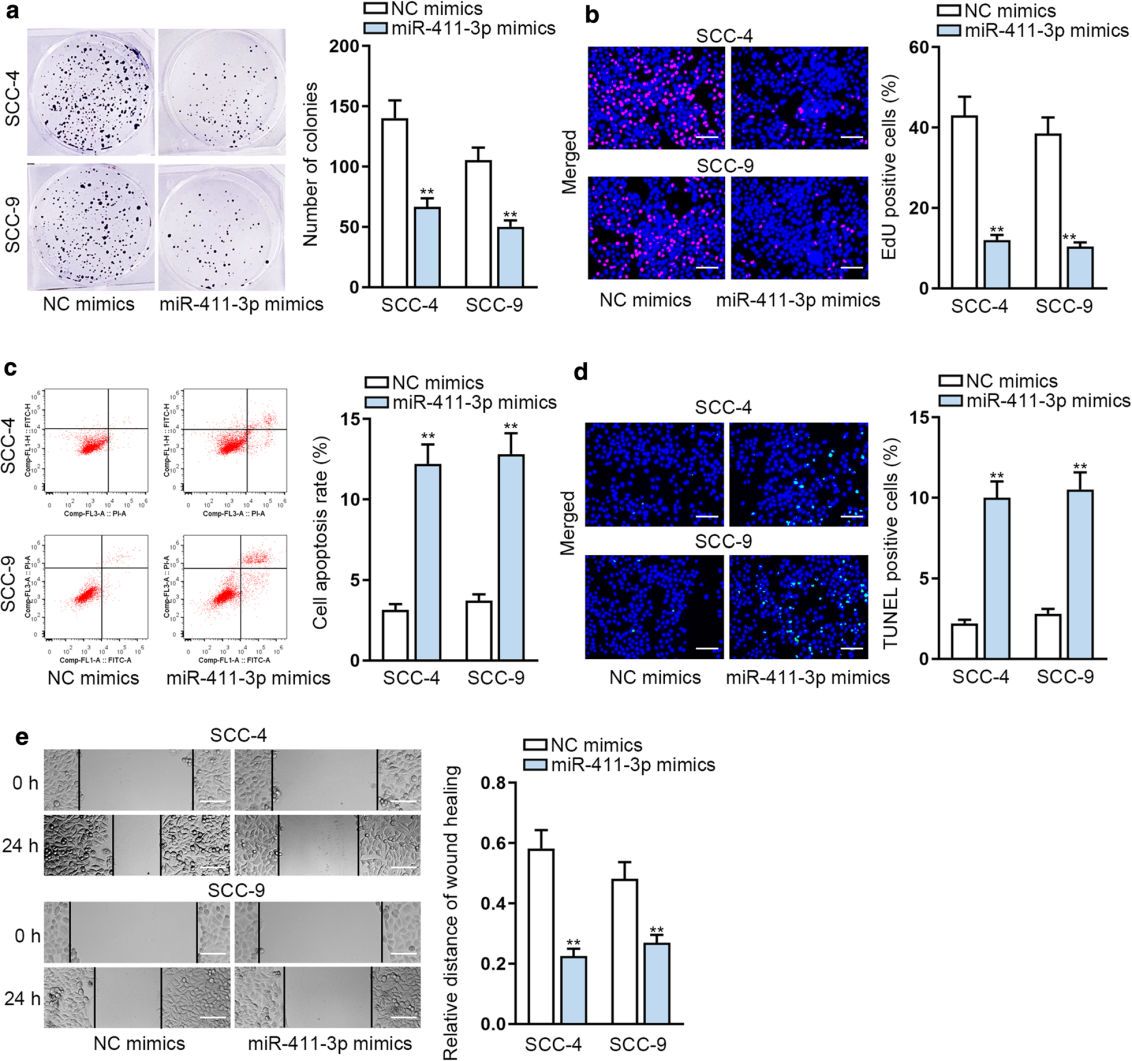
Upregulation of miR-411-3p represses cell proliferation and migration in OSCC. **a**, **b** Cell proliferation was estimated through colony formation and EdU experiments when miR-411-3p was overexpressed. **c**, **d** Flow cytometry and TUNEL experiments were implemented to measure cell apoptosis after overexpressing miR-411-3p. **e** Wound healing assays were adopted to test cell migration ability when miR-411-3p was subjected to upregulation. **P < 0.01

In order to search the role of miR-411-3p in OSCC, functional experiments were implemented. Firstly, colony formation and EdU assays indicated that overexpressing miR-411-3p suppressed the proliferation of SCC-4 and SCC-9 cells (Fig. 3a, b). Moreover, apoptosis of SCC-4 and SCC-9 cells was accelerated by miR-411-3p mimics through flow cytometry analysis and TUNEL assays (Fig. 3c, d). As illustrated in Fig. 3e, overexpression of miR-411-3p visibly reduced cell migration. Taken together, overexpression of miR-411-3p suppressed growth and migration in OSCC.

### NFAT5 is the downstream target of miR-411-3p in OSCC

**Fig. 4:**
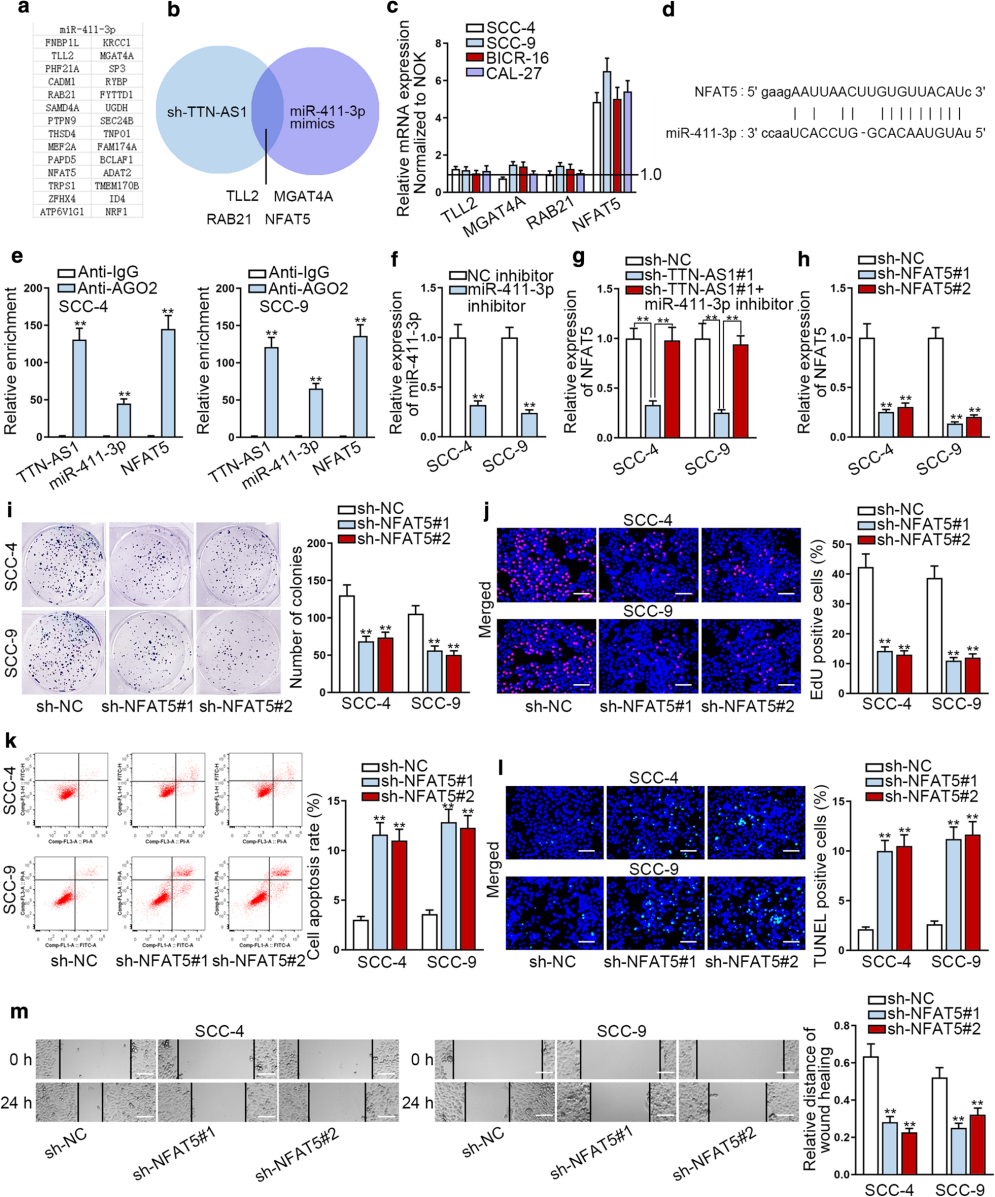
NFAT5 is a target gene of miR-411-3p in OSCC. **a** 28 mRNAs which had the binding site with miR-411-3p were predicted by starBase. **b** The qRT-PCR analysis was utilized to screen out the mRNAs which could be inhibited by NFAT5 depletion and miR-411-3p overexpression. **c** The expressions of TLL2, MGAT4A, RAB21 and NFAT5 in SCC-4 and SCC-9 cells through qRT-PCR. **d** The binding site of NFAT5 and miR-411-3p. **e** RIP assay was adopted to test the relationship between TTN-AS1, miR-411-3p and NFAT5. **f** The interference efficiency of miR-411-3p was tested by qRT-PCR analysis. **g** The expression of NFAT5 was detected when NFAT5 and miR-411-3p was silenced. **h** The interference efficiency of NFAT5 was tested by qRT-PCR analysis. **i**, **j** Cell proliferation was evaluated through colony formation and EdU experiments when NFAT5 was knocked down. **k**, **l** Cell apoptosis was measured through flow cytometry and TUNEL experiments after inhibiting NFAT5. **m** Wound healing assays were carried out for estimating cell migration after NFAT5 was subjected to inhibition. **P < 0.01

For the sake of further verifying ceRNA hypothesis, we searched the targets of miR-411-3p. Combining the searching results from miRmap, microT and PicTar databases, 28 candidate target genes were found under the condition (Program number: 4 programs) (Fig. 4a). Then, qRT-PCR assay was applied to detect the influence of miR-411-3p overexpression and TTN-AS1 inhibition on the levels of these mRNAs. The results displayed a significant down-regulation of 4 mRNAs (TLL2, MGAT4A, RAB21 and NFAT5) when miR-411-3p was overexpressed and TTN-AS1 was knocked down, while other mRNAs were almost unchanged (Fig. 4b). Then, we tested the expressions of TLL2, MGAT4A, RAB21 and NFAT5 in OSCC cells through qRT-PCR for further detection. We discovered that only NFAT5 displayed a high expression in OSCC cells (Fig. 4c). High level of NFAT5 was further determined in OSCC tissues compared to adjacent normal ones (Additional file 3: Fig. S2A). Thus, we selected NFAT5 to conduct the further experiments. Following, we discovered the binding site of NFAT5 and miR-411-3p from starBase (Fig. 4d). And RIP assays were implemented to evaluate the relationship of TTN-AS1, NFAT5 and miR-411-3p. The results showed that TTN-AS1, NFAT5 and miR-411-3p were enriched in Ago2, indicating that TTN-AS1/miR-411-3p/NFAT5 axis combined with RISC (Fig. 4e and Additional file 3: Fig. S2B). Then miR-411-3p was silenced and the interference efficiency was detected. We could observe that miR-411-3p expression exactly declined after inhibition (Fig. 4f). Following, we detected the expression of NFAT5 when TTN-AS1 and miR-411-3p were inhibited through qRT-PCR. Results indicated that NFAT5 expression could be hampered by TTN-AS1 depletion but then recovered by miR-411-3p inhibition (Fig. 4g and Additional file 3: Fig. S2C). It demonstrated that NFAT5 and TTN-AS1 were positively associated while NFAT5 and miR-411-3p were negatively correlated. Then we investigated the function of NFAT5 in OSCC cells. Firstly, we knocked down the expression of NFAT5 in SCC-4 and SCC-9 cells and tested the knockdown efficiency (Fig. 4h and Additional file 3: Fig. S2D). NFAT5 expression could be hampered effectively after knockdown. Then colony formation and EdU assays were carried out and the result indicated that silencing NFAT5 repressed the proliferation of SCC-4 and SCC-9 cells (Fig. 4i, j). Moreover, cell apoptosis capability was expedited by NFAT5 depletion in flow cytometry and TUNEL assays (Fig. 4k, l). Finally, wound healing assays indicated that silencing NFAT5 could hamper cell migration capability (Fig. 4m). Collectively, NFAT5 was a target gene of miR-411-3p in OSCC and it accelerated the progression of OSCC.

### TTN-AS1 promotes OSCC progression via miR-411-3p/NFAT5 axis

**Fig. 5:**
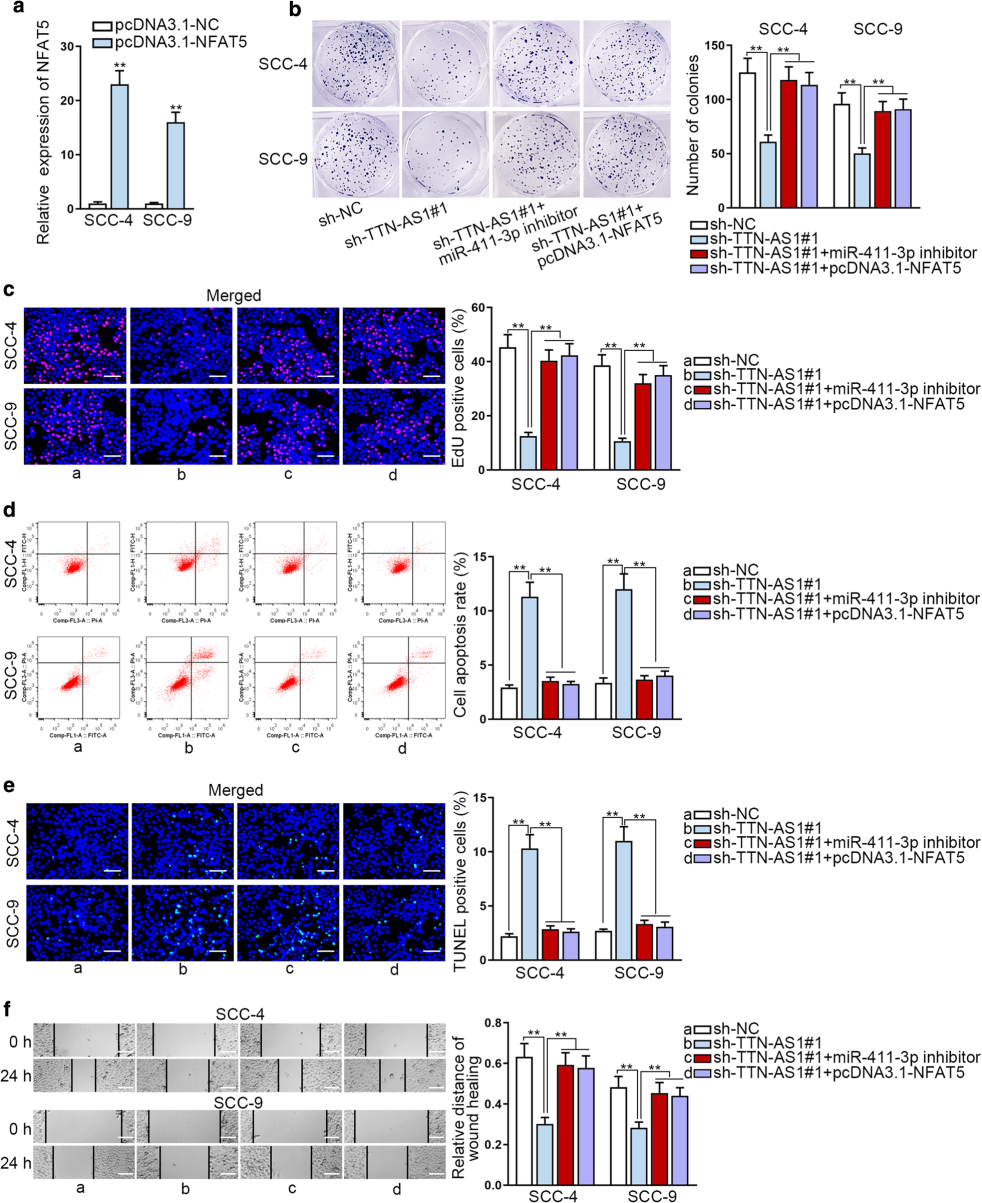
TTN-AS1 promotes OSCC progression via miR-411-3p/NFAT5 axis. **a** The qRT-PCR analysis was utilized to examine the overexpression efficiency of NFAT5 in SCC-4 and SCC-9 cells. **b**, **c** Cell proliferation capability in SCC-4 and SCC-9 cells was measured by colony formation and EdU assay in different groups. **d**, **e** Cell apoptosis was tested through flow cytometry and TUNEL assays in different groups. **f** Wound healing assays were implemented to detect the cell migration ability in different groups. **P < 0.01

For the sake of proving whether TTN-AS1 could accelerate OSCC progression via miR-411-3p/NFAT5 axis, rescue assays were implemented. Ahead of rescue assays, qRT-PCR was adopted to test the overexpression efficiency of NFAT5 in SCC-4 and SCC-9 cells. The results displayed that NFAT5 expression was visibly increased after transfecting with pcDNA3.1/NFAT5 (Fig. 5a). Next, we detected the mRNA and protein levels of NFAT5 in SCC-4 and SCC-9 cells after transfection. It was uncovered that NFAT5 levels decreased by TTN-AS1 depletion were rescued by the inhibition of miR-411-3p or the upregulation of NFAT5 (Additional file 3: Fig. S2E). Then colony formation and EdU rescue assays were conducted, we discovered that cell proliferation was hampered by TTN-AS1 depletion, but then it was recovered by NFAT5 overexpression or miR-411-3p inhibition (Fig. 5b, c). Through flow cytometry and TUNEL assays, we found that knockdown of miR-411-3p or upregulation NFAT5 could reverse the cell apoptosis ability which was accelerated by TTN-AS1 depletion (Fig. 5d, e). In the end, it was indicated through wound healing assay that the inhibited cell migration caused by knockdown of TTN-AS1 was restored by NFAT5 overexpression or miR-411-3p inhibition (Fig. 5f). Thus, we confirmed that TTN-AS1 promoted OSCC cell growth and migration by miR-411-3p/NFAT5 axis.

### TTN-AS1 promoted OSCC cell growth in vivo

**Fig. 6:**
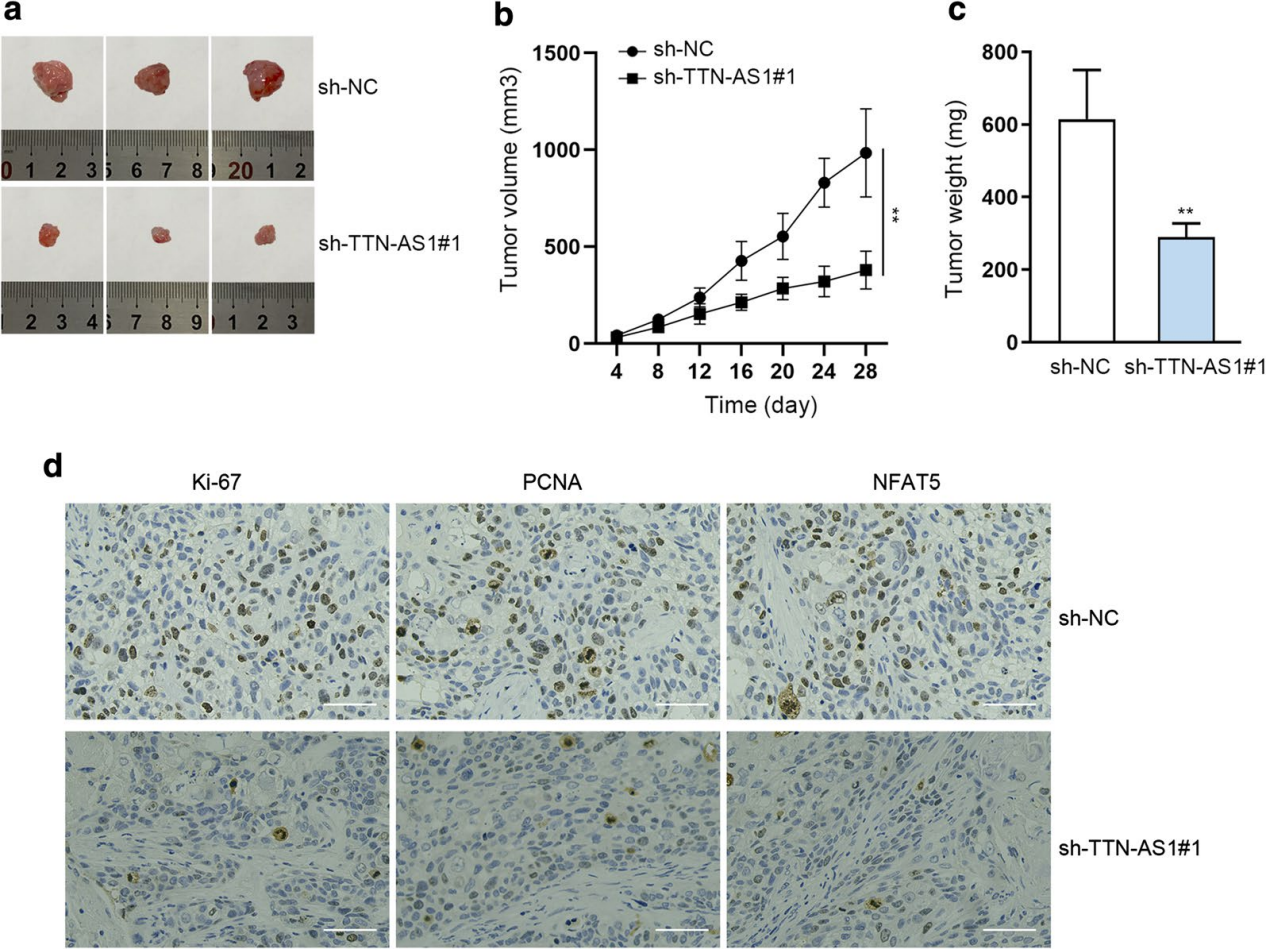
TTN-AS1 promoted OSCC cell growth in vivo. **a** Tumors removed from the mice injected with sh-NC-transfected cells or sh-TTN-AS1#1-transfected cells. **b**, **c** Volume and weight in different groups were measured. **d** IHC staining of tumor tissues collected from different groups with anti-Ki-67 and anti-PCNA. **P < 0.01

In vivo study was conducted to support above in vitro findings. We observed that tumor size, volume and weight in sh-NC group were all smaller than those in sh-TTN-AS1#1 group (Fig. 6a–c). Importantly, IHC staining indicated that silencing of TTN-AS1 caused a reduction in the positivity of Ki-67 and PCNA (Fig. 6d). All these experiments unveiled that TTN-AS1 promotes OSCC progression via miR-411-3p/NFAT5 axis.

## Discussion

Oral squamous cell carcinoma (OSCC) is a common squamous cell carcinoma of the head and neck. It has a relatively high incidence worldwide. As the regulatory functions of lncRNA in assorted cancers are constantly being explored, lots of lncRNAs have also been confirmed to play a crucial role in promoting the development of OSCC. For example, PLAC2 could promote cell growth through activating wnt/β-catenin pathway in OSCC [27]. CEBPA-AS1 was considered to correlate with the bad prognosis and it also could facilitate tumorigenesis through CEBPA/Bcl2 in OSCC [28]. Moreover, P4713 was reported to contribute to the malignant phenotypes of OSCC through activating the JAK/STAT3 pathway [29]. In our research, we investigated the functions of TTN-AS1 in OSCC. TTN-AS1 was a novel lncRNA and it served as the oncogene in lung adenocarcinoma [13]. In this study, TTN-AS1 was discovered to be highly expressed in OSCC cells. And TTN-AS1 depletion impaired cell proliferation and migration, but it accelerated cell apoptosis in OSCC. Overall, TTN-AS1 exerted the carcinogenic effect in OSCC.

MiRNAs are small RNAs with 22–24 nucleotides in length without ability of coding protein [30]. In recent years, an increasing number of evidences discovered that lncRNA could function as a crucial element of competing endogenous RNA (ceRNA) network by sponging miRNA to regulate mRNA, so as to take part in the regulation of cancer progression [31, 32]. For example, lncRNA ATB functioned as a ceRNA to expedite YAP1 through sponging miR-590-5p in malignant melanoma [33]. PAGBC acted as a sponge of miR‐133b and miR‐511 and accelerated gallbladder tumorigenesis [34]. AFAP1-AS1 could act as a ceRNA of miR-423-5p to expedite nasopharyngeal carcinoma progression [35]. In our research, we utilized bioinformatics tools to find the possible miRNA which could bind to TTN-AS1. After screening, miR-411-3p was selected. With the conduction of RIP and luciferase experiments, we proved that TTN-AS1 could act as ceRNA to sponge miR-411-3p in OSCC. MiR-411-3p was verified as the tumor suppressor gene in ovarian cancer, and it could restrain cell proliferation, migration and invasion of ovarian cancer [36]. Thus, we investigated the functions of miR-411-3p in OSCC. As we expected, miR-411-3p could repress cell proliferation and migration but accelerate cell apoptosis in OSCC. In short, our research confirmed that TTN-AS1 sponged miR-411-3p and overexpressing miR-411-3p could repress the progression of OSCC.

NFAT5 is a mRNA and it has been reported to be associated with several cancers. For example, NFAT5 was proved to conduce to the glycolytic phenotype rewiring and pancreatic cancer progression through transcription of PGK1 [37]. Moreover, NFAT5 cpuld also promote glioblastoma cell-driven angiogenesis through EGFL7 which was mediated via SBF2-AS1 and miR-338-3p [38]. In our research, we discovered that NFAT5 was highly expressed in OSCC cells. And based on the mechanism experiments, we also proved that NFAT5 was the target of miR-411-3p and overexpressing it could accelerate the progression of OSCC. Rescue experiment indicated that upregulation of NFAT5 could offset TTN-AS1 knockdown-mediated functions on the progression of OSCC, proving the functions of TTN-AS1/miR-411-3p/NFAT5 axis in OSCC.

## Conclusion

Taken together, TTN-AS1 could contribute to the progression of OSCC via miR-411-3p/NFAT5 axis, which may provide the new idea for the exploration of OSCC treatments.

## Supplementary information

**Additional file 1:** Sequence for all plasmids used in current study.

**Additional file 2: Figure S1** (A) TTN-AS1 expression in adjacent normal and tumor tissues was examined by qRT-PCR analysis. (B) CCK-8 assay was applied to analyze the viability of SCC-4 and SCC-9 cells transfected with sh-NC, sh-TTN-AS1#1 or sh-TTN-AS1#2. (C) The level of miR-411-3p was assessed in 50 pairs of OSCC tissues and adjacent normal tissues. (D) Agarose gel electrophoresis for the Ago2-RIP assay in Fig. 2F. **P < 0.01.

**Additional file 3: Figure S2** (A) NFAT5 expression in paired tissues obtained from 50 OSCC patients. (B) Agarose gel electrophoresis for the Ago2-RIP assay in Fig. 4E. (C) Protein level of NFAT5 in cells transfected with sh-NC, sh-TTN-AS1#1 or co-transfected with sh-TTN-AS1#1 and miR-411-3p inhibitor. (D) Protein level of NFAT5 in cells transfected with sh-NC, sh-NFAT5#1 and sh-NFAT5#2. (E) mRNA and protein level of NFAT5 in cells transfected with sh-TTN-AS1#1 was examined by qRT-PCR and western blot analyses after co-transfection with miR-411-3p inhibitor or pcDNA3.1/NFAT5. **P < 0.01.

## Data Availability

Not applicable.

## Abbreviations

OSCC: Oral squamous cell carcinoma
TTN-AS1: Titin antisense RNA 1
lncRNAs: Long non-coding RNAs
ceRNAs: Competing endogenous RNAs
miRNAs: microRNAs
mRNA: Messenger RNA
ATCC: American type culture collection
DMEM: Dulbecco’s modified Eagle’s medium
FBS: Fetal bovine serum
RIPA: Radioimmunoprecipitation assay
SDS-PAGE: Sulphate-polyacrylamide gel electrophoresis
PVDF: Polyvinylidene fluoride
RT-qPCR: RNA extraction and quantitative real-time polymerase chain reaction
HRP: Horseradish peroxidase
FISH: Fluorescence in situ hybridization
WT: Wild-type
Mut: Mutant
SD: Standard deviation
ANOVA: Analysis of variance
