# Supplementary figures and images for: LncRNA TTN-AS1 promotes the progression of oral squamous cell carcinoma via miR-411-3p/NFAT5 axis
Source: Cancer Cell Int. 2020 Aug 28;20:415. doi: 10.1186/s12935-020-01378-6 (PMC7453543; doi:10.1186/s12935-020-01378-6)

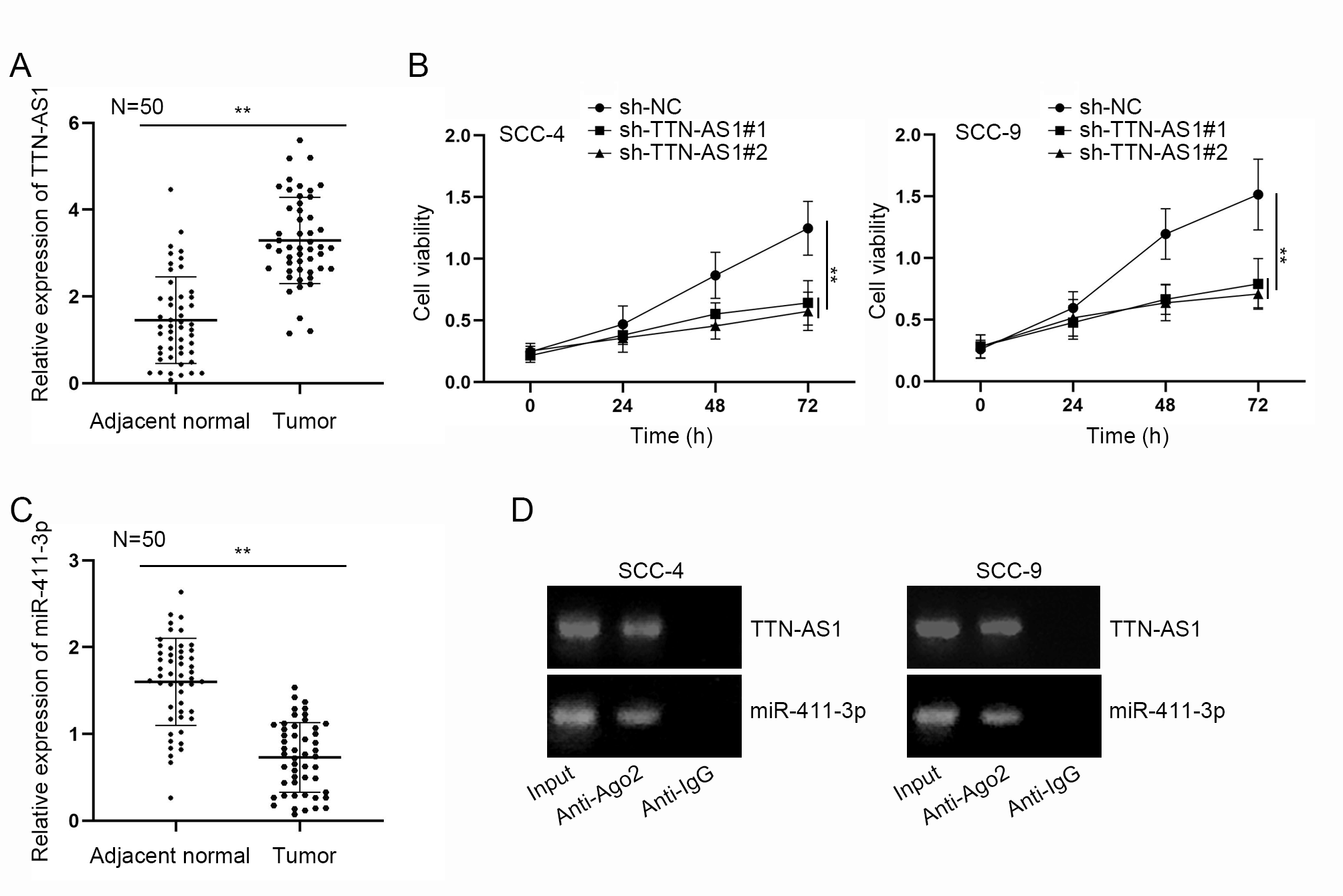

Supplement: Supplementary file 2 — Additional file 2: Figure S1 (A) TTN-AS1 expression in adjacent normal and tumor tissues was examined by qRT-PCR analysis. (B) CCK-8 assay was applied to analyze the viability of SCC-4 and SCC-9 cells transfected with sh-NC, sh-TTN-AS1#1 or sh-TTN-AS1#2. (C) The level of miR-411-3p was assessed in 50 pairs of OSCC tissues and adjacent normal tissues. (D) Agarose gel electrophoresis for the Ago2-RIP assay in Fig. 2F. **P < 0.01. [file 12935_2020_1378_MOESM2_ESM.tif]

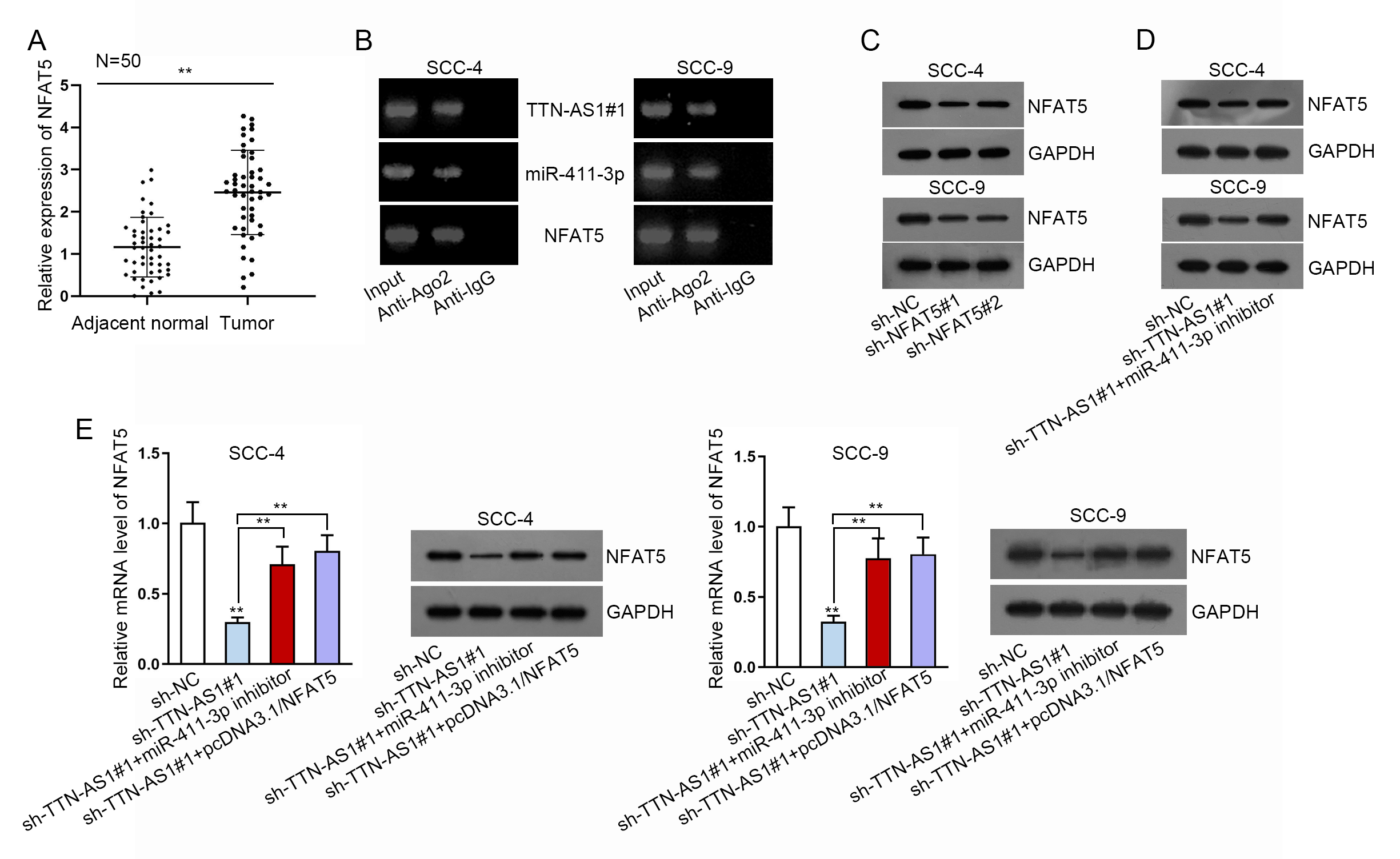

Supplement: Supplementary file 3 — Additional file 3: Figure S2 (A) NFAT5 expression in paired tissues obtained from 50 OSCC patients. (B) Agarose gel electrophoresis for the Ago2-RIP assay in Fig. 4E. (C) Protein level of NFAT5 in cells transfected with sh-NC, sh-TTN-AS1#1 or co-transfected with sh-TTN-AS1#1 and miR-411-3p inhibitor. (D) Protein level of NFAT5 in cells transfected with sh-NC, sh-NFAT5#1 and sh-NFAT5#2. (E) mRNA and protein level of NFAT5 in cells transfected with sh-TTN-AS1#1 was examined by qRT-PCR and western blot analyses after co-transfection with miR-411-3p inhibitor or pcDNA3.1/NFAT5. **P < 0.01. [file 12935_2020_1378_MOESM3_ESM.tif]
